# Negative effects on the development of *Chrysodeixis includens* and *Spodoptera cosmioides* fed by peanut plants inoculated with entomopathogenic fungi

**DOI:** 10.3389/ffunb.2022.968528

**Published:** 2023-01-26

**Authors:** Fernando Belezini Vinha, Luis Angel Chicoma Rojas, Cinara Ramos Sales, Natalia Sarmanho Monteiro Lima, Joacir Do Nascimento, Lucas Amoroso Lopes De Carvalho, Eliana Gertrudes De Macedo Lemos

**Affiliations:** ^1^ Department of Agricultural, Livestock and Environmental Biotechnology, Laboratory of Biochemistry and Plant Microorganisms, São Paulo State University (UNESP), School of Agricultural and Veterinary Sciences, Jaboticabal, Brazil; ^2^ Department of Agricultural Production Sciences, Laboratory of Microbial Biological Control of Arthropod Pests, School of Agricultural and Veterinary Sciences, São Paulo State University (UNESP), Jaboticabal, Brazil; ^3^ Department of Agricultural, Livestock and Environmental Biotechnology, Laboratory of Bioinformatics, School of Agricultural and Veterinary Sciences, São Paulo State University (UNESP), Jaboticabal, Brazil

**Keywords:** biological control (CB), entomopathogen, groundnut *(A. hypogaea L.)*, hypocreales, Lepidoptera

## Abstract

Recent studies have shown that entomopathogenic fungi, as endophytes, can have beneficial effects on plants, protecting them from defoliating insects. The potential of endophytic association by entomopathogenic fungi with the peanut crop has been little explored. In our study, we conducted experiments by inoculation of peanut seeds through a soil drench method with nine strains/species of entomopathogenic fungi of the genera *Metarhizium*, *Beauveria* and *Cordyceps*, subsequently these plants were consumed by two larval pests, *Chrysodeixis includens* and *Spodoptera cosmioides*. The parameters of larval growth rates, mortality, foliar consumption and larval period were observed during the development of larvae. In addition, the endophytic capacity of these fungi in peanut plants and their persistence in soil were investigated. In two replicate greenhouse trials for each larva, peanut plants were inoculated with fungi by the soil-drench method. We evaluated the performance of *C. includens* and *S. cosmioides* feeding on inoculated peanut plants starting at the 2nd larval instar. The larval and pupal weights of *C. includens* and *S. cosmioides* were significantly different among the fungal treatment groups, where insects feeding on control plants exhibited higher larval and pupal weights than insects feeding on treated plants. The differences in larval period showed that Control larvae pupated faster than the larvae fed on fungal-inoculated plants, fungal treatments had a larval period of 3 to 5 days more than the control. The mortality rates of *C. includens* and *S. cosmioides* were significantly different among the fungal treatment groups, insects fed on Control plants exhibited higher survival than insects fed on fungal-inoculated plants. The persistence of all *Metarhizium* fungi was higher in the soil compared to other fungi, and only *Metarhizium* and *B. bassiana* IBCB215 emerged from the phyllosphere of peanut plants. Although the fungus Cordyceps presented the worst performance among the fungal treatments. Overall, our results demonstrate the negative effects on the development of *C. includens* and *S. cosmioides* that were fed on fungal-inoculated peanut plants, the best results recorded were for *Metarhizium* strains and the fungus *B. bassiana* IBCB215.

## Introduction

The peanut (*Arachis hypogaea* L.) is an economically important oil seed crop that can be grown as a food resource, cover crop, green manure, forage, intercrop and hay (Akram et al., 2018). The peanut is important because it is the fourth most planted oilseed in the world, whose grains represent an important source of carbohydrates, protein and oil. In addition, it is rich in vitamins and amino acids and is considered a high-calorie food (Davis et al., 2016).

Brazil produced more than 700.5 mt of peanuts in 2021/22 and occupies the 11^th^ position among the world’s largest producers, being the second largest exporter in Latin America (USDA, 2021). The largest concentration of Brazilian production is in the southeast, with the state of São Paulo being the largest producer in the country (Conab, 2022).

Nevertheless, the high agricultural cost due to the need for frequent applications of pesticides for pest control puts the viability of peanut agribusiness at risk (Santos et al., 2013; Pirotta et al., 2017). Michelotto et al. (2015) estimate that approximately 30% of the total cost of production of a hectare is spent on the control of pests and diseases affecting the crop. In addition, frequent applications of pesticides have caused negative effects on the populations of natural enemies, leading to unbalanced agrosystems and pest population outbreaks (Moscardi et al., 2011).

Due to concerns about the influence of pesticides on the environment and human safety, it is necessary to push forward the development of environmentally friendly and economically viable and reliable strategies (Mantzoukas and Eliopoulos, 2020).

Among the main pests that occur in peanuts, the lepidopterous genera *Stegasta*, *Spodoptera*, *Chrysodeixis* and *Helicoverpa* are the most important (Michelotto et al., 2012; Pinto et al., 2020; Virk et al., 2021; Costa et al., 2022). In addition to lepidoptera, the peanut crop is severely attacked by thrips, especially the honeysuckle thrips *Enneothrips flavens* Molton (Lourenção et al., 2007; Calore et al., 2012; Michelotto et al., 2012).

Entomopathogenic fungi have emerged as a sustainable alternative in agroecosystems, as they can infect a large number of arthropods and maintain the pest population in balance through the field as a safe alternative to toxic chemical insecticides (Alves, 1998; Litwin et al., 2020). In Brazil, several national and multinational companies have been marketing entomopathogenic fungi as biopesticides for the control of major agricultural pests (Mascarin et al., 2019).

In addition to their use as biological insecticides, there is growing evidence that many species of entomopathogenic fungi can colonize the tissues of certain plants (Vega et al., 2008; Bamisile et al., 2018). Although only a few species have been reported as natural endophytes, there have been many successful attempts to artificially introduce the entomopathogenic fungi into plants using different techniques (Vega, 2018). This natural or artificial colonization can have several beneficial effects on plants, such as promotion of plant growth (Sasan and Bidochka, 2012), improved nutrient acquisition (Behie and Bidochka, 2014) and protection against phytopathogens and pests (Jaber and Enkerli, 2017; Vega, 2018).

Endophytic fungi can have significantly longer efficacy periods than nonendophytic organisms, since many are able to survive for at least the entire growing season of an annual crop (Harman et al., 2008). Endophytic strains of fungi, which colonize trees, can settle on the shoots, roots, or stems of perennial plants (Worapong et al., 2002; Hanada et al., 2008). Therefore, the use of entomopathogenic fungi as endophytes provides a new alternative for the biological control of pests and phytopathogens (Vidal and Jaber, 2015).

Most studies with entomopathogenic endophytic fungi have been conducted on annual and perennial crops of agronomic importance; however, studies with peanut plants are nonexistent (Vega, 2018; Mantzoukas et al., 2022). In this context, our study evaluated the effects of several species of entomopathogenic fungi on the development and plant consumption of *Spodoptera cosmioides* and *Chrysodeixis includens* in inoculated peanut plants.

## Materials and methods

### Fungal strains and inoculum preparation

Nine isolates of the fungi *B. bassiana*, *M. anisopliae* and *C. fumosorosea* were obtained from the Mycological Collection of EMBRAPA, CFI - Invertebrate Fungi (CG, Brasília, DF, Brazil) and Instituto Biológico (IBCB, Campinas, SP, Brazil) and used in bioassays (see **Table 1**).

**Table 1:** Origin of entomopathogenic fungi used in the bioassays.

Isolates	Strains	Origin	Host
IBCB215	*Beauveria bassiana*	Ribeirão Preto - SP	Soil
IBCB66	*Beauveria bassiana*	São José do Rio Pardo SP	*Hypothenemus hampei*
CG1126	*Metarhizium brunneum*	Flores da Cunha - RS	*Eurhizococcus brasiliensis*
CG814	*Metarhizium humberi*	Dom Aquino - MT	*Scaptocoris castanea*
CG1123	*Metarhizium alvesii*	Quixeré - CE	Soil
IBCB425	*Metarhizium anisopliae*	Iporanga - SP	Soil
IBCB348	*Metarhizium anisopliae*	Araras - SP	*Mahanarva fimbriolata*
IBCB 130	*Cordyceps fumosorosea*	Florínia-SP	Soil
IBCB 867	*Cordyceps fumosorosea*	Palmeiras de Goias-GO	*Helicoverpa armigera*
Control	-	-	*-*

CG = Mycological Collection of EMBRAPA, CFI - Invertebrate Fungi, Brasília, DF, Brazil.

IBCB = “Oldemar Cardim Abreu” Collection of Entomopathogen Cultures, Instituto Biológico, Campinas, São Paulo, Brazil.

Fungal isolates were grown on Petri dishes with potato-dextrose-agar (PDA; KASVI) medium and incubated in a B.O. D (Bio-Oxygen Demand) incubator in the dark at 26 ± 2°C for 10 days.

Subsequently, solid fermentation was performed to increase the amount of inoculum used for the experiments. For solid fermentation, 250 ml borosilicate glass bottles (Schott bottles) containing 50 g of parboiled rice were used. Initially, the substrate was hydrated with 100 ml of distilled water and kept at room temperature for 50 min. Then, the rice was sieved to remove excess water and returned to the Schott bottles to be autoclaved at 120°C for 20 min.

The inoculum of each fungal isolate was prepared by scraping the conidia present in the PDA culture medium and diluting with sterilized distilled water with 0.01% (v/v) Tween 80^®^. Inoculation of Schott bottles was performed in an aseptic laminar flow chamber by adding 5 ml of conidia suspension of each isolate, and then all flasks were kept in B.O.D. in the dark at 26± 2°C for 10 days.

After the proliferation of fungal isolates, a conidial suspension was prepared by washing the rice grains with 150 ml of 0.01% (v/v) Tween 80^®^ in each flask, and the conidial concentration was adjusted to 1 x 10^8^ conidia/ml by counting in a Neubauer hemocytometer (KASVI) with an optical microscope (LEICA DMLB).

To confirm the viability of the fungi used in the experiments, conidia germination was determined by the direct counting method in 4 ml of PDA corrected with 0.001% (v/v) Derosal^®^ 500 SC (Carbendazim, Bayer CropScience, SP, Brazil) in Rodac^®^ plates incubated with suspensions of 1x10^6^ conidia/ml for each fungal species (Oliveira et al., 2015).

Subsequently, the Petri plates were kept in B.O.D. for 22 h in the dark at 26 ± 2°C. The number of germinated and nongerminated conidia was quantified under an optical microscope, estimating the percentage of germinated conidia. The suspensions were used only if germination rates were greater than 90%.

### Obtaining neonatal larvae

The larvae of *C. includens* and *S. cosmioides* were obtained from the Insect Biology Laboratory of the Department of Entomology and Acarology of ESALQ-USP, Piracicaba, State of São Paul, Brazil. The eggs of both species were placed in plastic jars (500 ml) containing moist filter paper and kept in a B.O.D. chamber at 26 ± 1°C for 12 h of photophase. First instar neonates were fed on control (uninoculated) peanut until they reached the 2^nd^ instar to acclimate larvae to feed on live plants versus artificial diet.

### Peanut seed inoculation with entomopathogenic fungi

Peanut seeds of the variety IAC OL-3 were obtained from COPLANA - Agroindustrial Cooperative, Jaboticabal, SP, Brazil. The seeds were transferred to plastic pots (0.7 L) containing a nonsterilized soil mixture of clay loam soil and sand (2:1). Four seeds per cup were sown at a depth of 2 cm.

The inoculation of fungal isolates and the control treatment were performed by direct application of the conidia suspension on the seed (soil drenching) according to the methodology of Parsa et al. (2013). The inoculation of each treatment was performed by applying 2 ml of the suspension with 1 x 10^8^ conidia/ml on the seed after sowing. The control treatment was inoculated with 2 ml of sterile aqueous solution with 0.01% (v/v) Tween 80^®^ without adding fungal inoculum.

All inoculated plants were grown in a greenhouse for 30 days at ~27 ± 3°C, with a natural photoperiod (10.5 to 13.5 hours) for the duration of the experiment. Pots were placed in a completely randomized design, watered as needed and fertilized biweekly with 3 g/dm³ of Osmocote^®^ slow release pelletized fertilizer (N; P_2_O_5_; K_2_O - 14-14-14) per pot.

### *S. cosmioides* and *C. includens* developmental test on peanut plants inoculated with entomopathogenic fungi

We conducted two experiments, repeated twice in time, for each larva to check the effects of nine entomopathogenic fungi, *Metarhizium*, *B. bassiana* and *C. fumosorosea*, on the survival and development of *S. cosmioides* and *C. includens*. *S. cosmioides* larval infestation times were March 5, 2020, for the first assay and August 15^th^, 2021, for the second assay. *C. includens* larvae were set up on April 3, 2020, and September 21, 2021, for the first and second assays, respectively.

**Figure 1:**
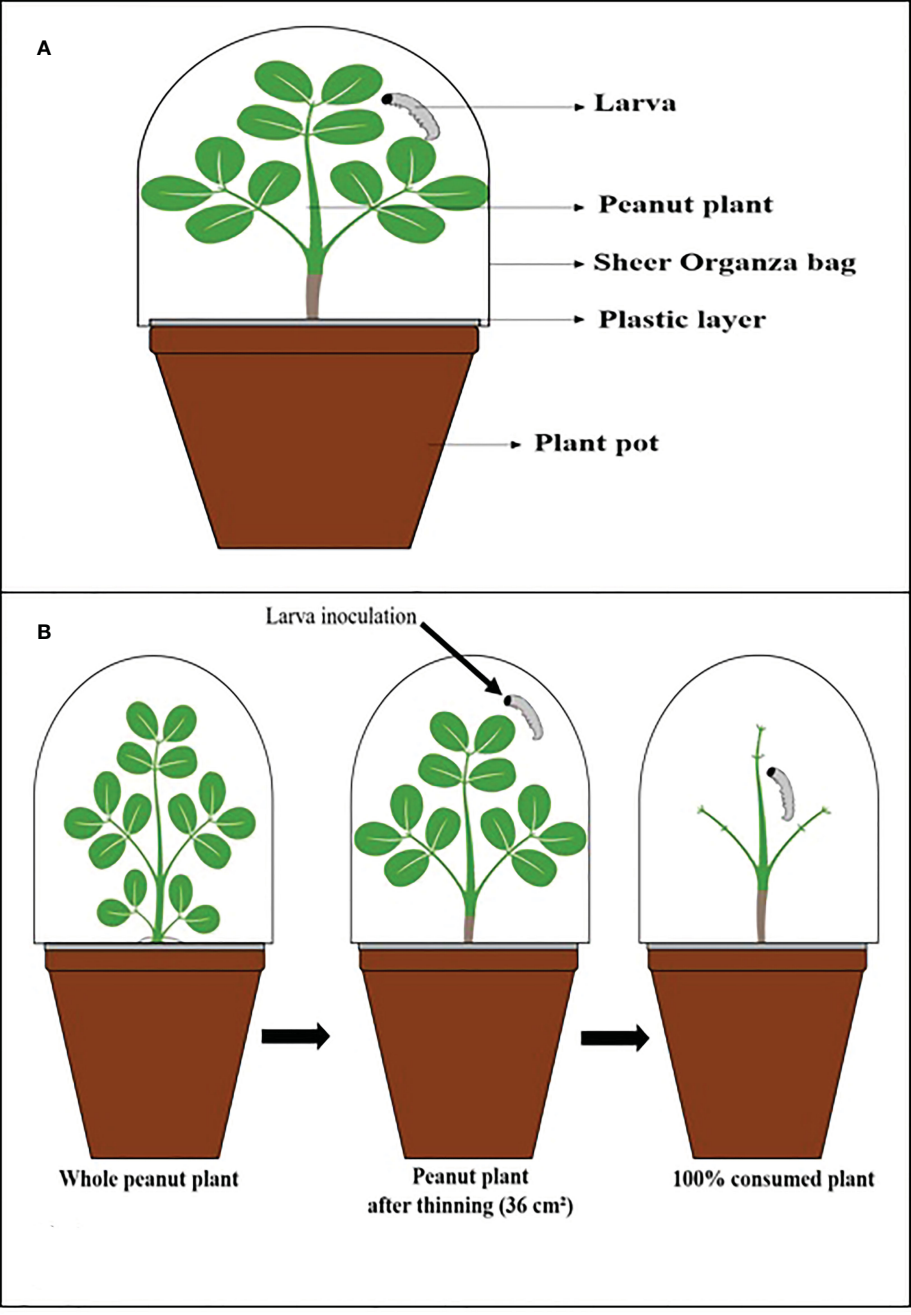
Illustrative scheme. **(A)** The vessel used in the experiments. **(B)** Thinning process of peanut plants, inoculation, and after being consumed by the larva.

For each experiment, 30-day-old peanut plants were inoculated with the entomopathogenic fungi described in **Table 1** and the control treatment, and in each pot, a plastic layer was placed on the soil surface to isolate the larvae by direct contact with the conidia in the soil. It was necessary to avoid contamination by conidia to accurately assess the indirect effects of leaf and stem consumption by larvae (**Figure 1**).

Each plant was infested with a 2^nd^ instar larva and covered with a sheer Organza bag (7 in x 9 in: 0.3 oz) to prevent larvae from escaping and allow airflow. The *C. includens* larvae were observed for 27 days and *S. cosmioides* for 30 days because each larval species has a different developmental period starting from the first instar.

Each larva within each plant was considered a repetition, and each treatment had 30 repetitions, totaling 30 infested pots per treatment. Larvae were weighed every 3 days on a high-precision scale. Mortality was recorded daily, and days to pupation, pupal weight and foliar consumption from control and treated plants were also recorded during both greenhouse trials.

If the plant reached a high level of defoliation, it was replaced by a new plant of the same treatment to continue the experiment until the larvae reached the pupal stage. For the replacement of fungal-inoculated peanut plants, new plants were sown throughout the experiments. The sowing dates of the new lots were consecutive, to always have plants of the same age and not lack food for both larvae.

The estimate of leaf area consumed was analyzed with LeafByte mobile application (Getman-Pickering et al., 2020). Twenty-five plants 30 days old were collected and pricked, leaving only 3 pairs of 4 leaflets, and the leaves were measured to generate an estimate of leaf area for each plant.

The average was determined at 36.6 cm² for each plant. The entire consumption of a plant was considered when the larvae ate all the leaves (**Figure 1**), and then it was replenished with a new inoculated plant with the same age characteristics and fungal inoculation.

### Colonization of entomopathogenic fungi on peanut plants and soil

Peanut plants inoculated with fungal treatments were whole washed in distilled water to remove surface dirt on leaves and the soil at the roots 30 days after inoculation. Subsequently, the plant material (roots, stems and leaves) was cut with scissors into fragments.

For each treatment, 5 plants were randomly selected, totaling 60 plant fragments per treatment (20 roots, 20 stems and 20 leaves). These samples were superficially sterilized by immersion in 70% ethanol for 1 min, 1% sodium hypochlorite (NaOCl) for 2 min, and 70% ethanol for 1 min again, rinsed three times in sterile distilled water and dried on sterile filter paper. Sterilization efficacy was confirmed by plating 100 μl of the last rinse water on PDA media, and the absence of fungal or bacterial growth was considered indicative of a successful sterilization technique (Parsa et al., 2013), and each leaf section was printed on PDA media before and after sterilization (Greenfield et al., 2016).

Plant samples were then individually placed in Petri dishes (90×15 mm) containing 20 ml of PDA with 0.5 g/L cycloheximide, 0.2 g/L chloramphenicol, 0.5 g/L Dodine (65%) and 0.01 g/L Crystal Violet (Behie et al., 2015). Petri dishes were incubated in the dark at 24°C for 15 days. Prepared suspensions of the soil where the plants grew were also seeded on the same selective medium at the following four concentrations after serial dilution in distilled water + 0.05% Tween 80^®^: 1×10, 1×10^-1^, 1×10^-2^, and 1×10^-3^.

Petri dishes were incubated in the dark at 24°C for 15 days, and the presence of colonies (CFU) was quantified at each concentration after the incubation period. The presence of entomopathogenic fungi in plant tissues and diluted soil was confirmed by observing the growth of colonies of each fungal species on the culture medium and observing the reproductive structures under an optical microscope (morphological visualization), according to the taxonomic keys described by Humber (2012). The colonization frequency was estimated as the number of fragments colonized by entomopathogenic fungi in relation to the total number of plant fragments and expressed as a percentage.

### Statistical analysis

For larval weight, period, and foliar consumption, as well as per pupal weight, homogeneity of variance was performed using the Kolmogorov-Smirnov test, which showed that the data were normally distributed. For the mortality variable, the homogeneity of variance test was performed using the Shapiro-Wilk test.

To assess *S. cosmioides* and *C. includens* mortality, larval weight, pupal weight, larval period and larval foliar consumption, an analysis of variance (ANOVA) was performed in the software R Studio^®^ (library used “ExpDes.pt”, “agricolae” “nortest”) in a completely randomized design (CRD) (R Core Team, 2020). When a significant result was detected, the means were compared using Tukey’s test at 5% probability for mortality and Scott-Knott’s test at 5% probability for all other variables.

To compare the permanence of fungi, the quantification of colony forming units (CFU) per gram of soil was used. The log-transformed values ​​were submitted to one-way ANOVA, considering a confidence level of 95%. Given the existence of differences, the means of the groups were compared pairwise using the Tukey honest significant difference (Tukey-HSD) test at a 5% significance level (p value < 0.05).

Statistical tests were conducted in R software (R Core Team, 2020) using native functions and the “HSD.test” function of the “agricolae” package (Mendiburu, 2021). The graphical representation of the comparison of means was obtained with the “ggplot2” package (Wickham, 2016).

## Results

### *S. cosmioides* and *C. includens* larval and pupal weights

The weight of *S. cosmioides* larvae was recorded for 27 days and *C. includens* for 24 days, but due to discrepancies in weight values, delays in larval development and occasional deaths, analyses of larval weights were made 15 days after the experiments were installed. The weights of *S. cosmioides* and *C. includens* larvae fed on fungal-inoculate plants differed from the control, with the *Metarhizium* strains (CG814, CG1123, CG1126 and IBCB348) causing the lowest larval weights for both insect species (see **Table 2**).

**Table 2:** *S. cosmioides* and *C. includens* larval weights after feeding for 15 days on peanut plants inoculated with entomopathogenic fungi and uninoculated control.

Treatment	Larval weight on 15^th^ day (mg)	
	*Spodoptera cosmioides*	*Chrysodeixis includens*
Control	238.56 ± 5.30 a	261.04 ± 2.04 a
*Beauveria bassiana* **IBCB66**	191.35 ± 5.20 c	231.42 ± 2.41 b
*Cordyceps fumosorosea* **IBCB130**	207.59 ± 3.67 b	231.39 ± 4.80 b
*Beauveria bassiana* **IBCB215**	189.57 ± 7.23 c	207.81 ± 3.55 c
*Cordyceps fumosorosea* **IBCB867**	192.15 ± 4.12 c	192.25 ± 4.55 d
*Metarhizium anisopliae* **IBCB425**	180.19 ± 5.70 c	192.11 ± 3.34 d
*Metarhizium anisopliae* **IBCB348**	173.44 ± 5.91 d	188.41 ± 4.68 d
*Metarhizium brunneum* **CG1126**	161.11 ± 4.93 d	179.65 ± 4.88 e
*Metarhizium alvesii* **CG1123**	163.90 ± 3.77 d	176.15 ± 4.16 e
*Metarhizium humberi* **CG814**	163.96 ± 5.99 d	173.32 ± 4.59 e
**p value**	2.2 x 10 ^-16^	2.2 x 10 ^-16^
**Normality Kolmogorov-Smirnov**	0.09107	0.6996

Means followed by the same letter are not significantly different (Scott-Knott 5%).

For the *S. cosmioides* trial, a maximum weight of 238.56 ± 5.30 mg and a minimum weight of 161.11 ± 4.93 mg were recorded, corresponding to the control and CG1126 treatments, respectively. For the *C. includens* assay, the maximum and minimum weights were 261.04 mg and 173.32 ± 4.59 mg, respectively, corresponding to the treatment with strain CG814 (see **Table 2**).

In both assays, there were significant differences, and their probabilities of significance (p value) were 2.2 x 10 ^-16^ for both larvae. The normality values of the data, calculated by the Kolmogorov-Smirnov test, were 0.09107 for *S. cosmioides* and 0.6996 for *C. includens*.

All pupal weight treatments of both larvae were significantly different from the control, with the exception of IBCB130 and CG1123, which had no significant differences from the control for *C. includens* treatments. *S. cosmioides* individuals from fungi-inoculated treatments (CG1123, CG1126, IBCB215 and IBCB425**)** had the lowest pupal weights (see **Table 3**).

**Table 3:** *S. cosmioides* and *C. includens* pupal weights after feeding on peanut plants inoculated with entomopathogenic fungi and uninoculated control.

Treatment	Pupal weight (mg)	
	Spodoptera cosmioides	Chrysodeixis includens
*Metarhizium anisopliae* **IBCB425**	281.69 ± 2.58 c	273.97 ± 1.55 a
*Cordyceps fumosorosea* **IBCB130**	289.67 ± 1.93 b	272.07 ± 2.02 a
*Metarhizium alvesii* **CG1123**	280.77 ± 2.33 c	271.88 ± 1.30 a
*Cordyceps fumosorosea* **IBCB867**	286.14 ± 1.86 b	269.84 ± 2.01 b
*Metarhizium anisopliae* **IBCB348**	282.8 ± 2.50 c	269.67 ± 1.26 b
*Beauveria bassiana* **IBCB215**	281.16 ± 2.60 c	264.55 ± 2.23 b
*Metarhizium humberi* **CG814**	285.94 ± 2.13 b	268.52 ± 0.71 b
*Metarhizium brunneum* **CG1126**	278.92 ± 2.77 c	268.51 ± 0.99 b
*Beauveria bassiana* **IBCB66**	292.56 ± 2.35 b	266.66 ± 2.16 b
**Control**	327.26 ± 3.49 a	268.74 ± 2.00 b
**p value**	8.51 x 10^-45^	0.015023
**Normality Kolmogorov-Smirnov**	0.1055	0.5418

Means followed by the same letter are not significantly different (Scott-Knott 5%).

The lowest pupal weights for *C. includens* individuals with fungi inoculated treatments were IBCB215, IBCB66, CG1126 and CG814. The fungi *B. bassiana* IBCB215 and *Metarhizium* CG1126 were observed to show the significantly lowest pupal weight for both larvae *S. cosmioides* and *C. includens*.

The maximum and minimum average weights of *S. cosmioides* pupae were 327.26 ± 3.49 mg and 278.92 ± 2.77 mg, corresponding to the CG1126 and control treatments, respectively. However, *C. includens* treatments IBCB425 and IBCB215 recorded a weight of 273.97 ± 1.55 mg (max.) and 264.55 ± 2.23 mg (min.) (see **Table 3**). The probability values (p values) for the *S. cosmioides* and *C. includens* tests were 8.51 x 10^-45^ and 0.015023, respectively, and the normality of the data, according to the Kolmogorov-Smirnov test at 5%, was 0.1055 and 0.5418 for *S. cosmioides* and *C. includens*, respectively.

### *S. cosmioides* and *C. includens* larval period and foliar consumption

The larval periods of the *S. cosmioides* and *C. includens* treatments were significantly different from the larval period of the control, with the exception of IBCB130, which had no significant difference from the *S. cosmioides* control treatment (see **Table 4**).

**Table 4:** *S. cosmioides* and *C. includens* larval period after feeding on peanut plants inoculated with entomopathogenic fungi and uninoculated control.

Treatment	Larval period (days)	
	*Spodoptera cosmioides*	*Chrysodeixis includens*
*Metarhizium alvesii* **CG1123**	28.44 ± 0.29 a	25.60 ± 0.27 a
*Metarhizium humberi* **CG814**	28.32 ± 0.30 a	25.81 ± 0.25 a
*Metarhizium brunneum* **CG1126**	28.07 ± 0.28 a	24.84 ± 0.30 b
*Beauveria bassiana* **IBCB215**	27.26 ± 0.32 b	23.36 ± 0.36 c
*Metarhizium anisopliae* **IBCB348**	27.09 ± 0.35 b	24.50 ± 0.22 b
*Cordyceps fumosorosea* **IBCB867**	26.4 ± 0.19 c	23.87 ± 0.38 c
*Beauveria bassiana* **IBCB66**	26.35 ± 0.19 c	21.51 ± 0.31 d
*Metarhizium anisopliae* **IBCB425**	26.25 ± 0.22 c	23.48 ± 0.31 c
*Cordyceps fumosorosea* **IBCB130**	25.67 ± 0.27 d	21.87 ± 0.43 d
**Control**	24.97 ± 0.31 d	20.30 ± 0.24 e
**p value**	2,48 x 10^-23^	2,20 x 10^-16^
**Normality Kolmogorov-Smirnov**	0.0661	0.1240

Means followed by the same letter are not significantly different (Scott-Knott 5%).

Pairwise comparisons showed that control larvae pupated faster than the larvae fed on fungal-inoculated plants. The *Metarhizium* fungi treatments CG1123, CG814 and CG1126 had the longest *S. cosmioides* and *C. includens* larval periods. The fungi *Metarhizium* treatments CG1123 and CG814 showed the significantly longest larval delay for both larvae *S. cosmioides* and *C. includens*.

The *S. Cosmioides* treatments CG1123 and Control recorded the maximum (28.44 ± 0.29 days) and minimum (24.97 ± 0.31 days) larval periods; thus, there was a larval delay of approximately 3 days for fungal-inoculated plants in comparison to the control (see **Table 4**). For the *C. includens* treatments CG814 and Control, the maximum (25.81 ± 0.25 days) and minimum (20.30 ± 0.24 days) larval periods were recorded, exhibiting a larval delay of approximately 5 days for fungal-inoculated plants in comparison to the control.

The mean area of leaves consumed by both larvae differed significantly among treatments (*S. cosmioides*, p = 2.2 x 10 ^-16^ and *C. includens*, p = 0.000176) (see **Table 5**). The control and CG814 treatments for the *S. cosmioides* and *C. includens* assays recorded the maximum (261.05 ± 5.76 cm²; 165.06 ± 6.43 cm²) and minimum (1^st^ = 173.32 ± 7.94 cm²; 122.24 ± 7.93 cm²) leaf consumption values, respectively (see **Table 5**).

**Table 5:** Cumulative foliar consumption (cm²) of *S. cosmioides* and *C. includens* fed with peanut plants inoculated with entomopathogenic fungi and uninoculated control.

Treatment	Foliar consumption (cm²)	
	*Spodoptera cosmioides*	*Chrysodeixis includens*
**Control**	261.05 ± 5.76 a	165.06 ± 6.43 a
*Beauveria bassiana* **IBCB66**	231.42 ± 5.29 b	143.83 ± 7.68 a
*Cordyceps fumosorosea* **IBCB130**	231.40 ± 4.82 b	150.06 ± 6.02 a
*Beauveria bassiana* **IBCB215**	207.81 ± 7.14 c	122.61 ± 9.95 b
*Cordyceps fumosorosea* **IBCB867**	192.26 ± 5.40 d	142.74 ± 6.79 a
*Metarhizium anisopliae* **IBCB425**	192.12 ± 6.58 d	154.45 ± 6.63 a
*Metarhizium anisopliae* **IBCB348**	188.42 ± 7.61 d	139.81 ± 7.29 a
*Metarhizium brunneum* **CG1126**	179.65 ± 7.38 e	127.00 ± 8.20 b
*Metarhizium alvesii* **CG1123**	176.15 ± 7.75 e	126.63 ± 8.37 b
*Metarhizium humberi* **CG814**	173.32 ± 7.94 e	122.24 ± 7.93 b
**p value**	2.2 x 10 ^-16^	0.000176
**Normality Kolmogorov-Smirnov**	0.4032	00.69

Means followed by the same letter are not significantly different (Scott-Knott 5%).

The normality values of the data, calculated by the Kolmogorov-Smirnov test, were 0.4032 for the *S. cosmioides* test and 00.69 for the *C. includens* test. The fungal *Metarhizium* strains CG1126, CG1123, and CG814 presented lower foliar consumption by both larval species.

### S. cosmioides and C. includens mortality

Comparisons showed that control individuals lived longer on average than the individuals on fungal-treated plants. *Metarhizium* strains CG1126, CG814, CG1123 and *B. bassiana* IBCB215 caused the highest mortalities on both insect species (see **Table 6**). The highest mortality rates for both larvae were reported for the CG814 treatment, with 50 ± 2.82% for *S. cosmioides* and 38 ± 5.93% for *C. includens*.

**Table 6:** Mortality (%) of *S. cosmioides* and *C. includens* fed peanut plants inoculated with entomopathogenic fungi and the uninoculated control.

Treatment	Mortality (%)	
	*Spodoptera cosmioides*	*Chrysodeixis includens*
*Metarhizium brunneum* **CG1126**	46 ± 5.36 ab	38 ± 5.21 a
*Metarhizium humberi* **CG814**	50 ± 2.82 a	38 ± 5.93 a
*Metarhizium alvesii* **CG1123**	46 ± 4.56 ab	34 ± 3.57 ab
*Beauveria bassiana* **IBCB215**	34 ± 4.56 abc	34 ± 6.69 ab
*Metarhizium anisopliae* **IBCB348**	32 ± 4.38 abc	18 ± 5.93 abc
*Beauveria bassiana* **IBCB66**	16 ± 3.57 cd	18 ± 1.78 abc
*Cordyceps fumosorosea* **IBCB867**	20 ± 2.82 cd	18 ± 3.34 abc
*Metarhizium anisopliae* **IBCB425**	28 ± 4.38 bcd	12 ± 5.21 bc
*Cordyceps fumosorosea* **IBCB130**	14 ± 4.56 cd	8 ± 5.21 c
**Control**	8 ± 3.34 d	6 ± 2.19 c
**p value**	5.12 x 10^-08^	7.31 x 10 ^-05^
**Normality Shapiro-Wilk**	0.1912	0.06140

Means followed by the same letter are not significantly different (Tukey 5%).

The lowest mortality rate in fungal treatments was recorded in IBCB130, 14 ± 4.56% for *S. cosmioides* and 8 ± 5.21% for *C. includens* (see **Table 6**). The probability of significance (p value) for *S. cosmioides* and *C. includens* trials was 5.12 x 10^-08^ and 7.31 x 10 ^-05,^ respectively. Additionally, the normality of the data was estimated using the Shapiro-Wilk test, obtaining values of 0.1912 and 0.06140.

### Occurrence of entomopathogenic fungi in soil and peanut plants

*Metarhizium* (CG814, CG112, CG11236, IBCB348) and *B. bassiana* IBCB215 isolates became endophytic with relatively low colonization levels at 30 days after peanut seed inoculation (see **Table 7**). For *Metarhizium*, roots and leaves were colonized, while *B. bassiana* colonized stems and leaves (see **Table 7**). None of the target fungi were recovered from the plant tissue in the control treatment, *C. fumosorosea* isolates (IBCB 130, IBCB 867) and *B. bassiana* IBCB66. Occasionally, other unidentified fungi were grown from the plant tissues but with no apparent relationship to the treatment.

**Table 7:** Occurrence (%) of entomopathogenic fungi in peanut plant fragments at 30 days after inoculation.

S. cosmioides and C. includens			
Treatments	Root (%)	Stem (%)	Leaf (%)
*Beauveria bassiana* **IBCB215**	0	20	10
*Beauveria bassiana* **IBCB66**	0	0	0
*Metarhizium brunneum* **CG1126**	25	0	10
*Metarhizium humberi* **CG814**	25	0	10
*Metarhizium alvesii* **CG1123**	20	0	15
*Metarhizium anisopliae* **IBCB425**	10	0	0
*Metarhizium anisopliae* **IBCB348**	15	0	5
*Cordyceps fumosorosea* **IBCB 130**	0	0	0
*Cordyceps fumosorosea* **IBCB 867**	0	0	0
**Control**	0	0	0

**Figure 2:**
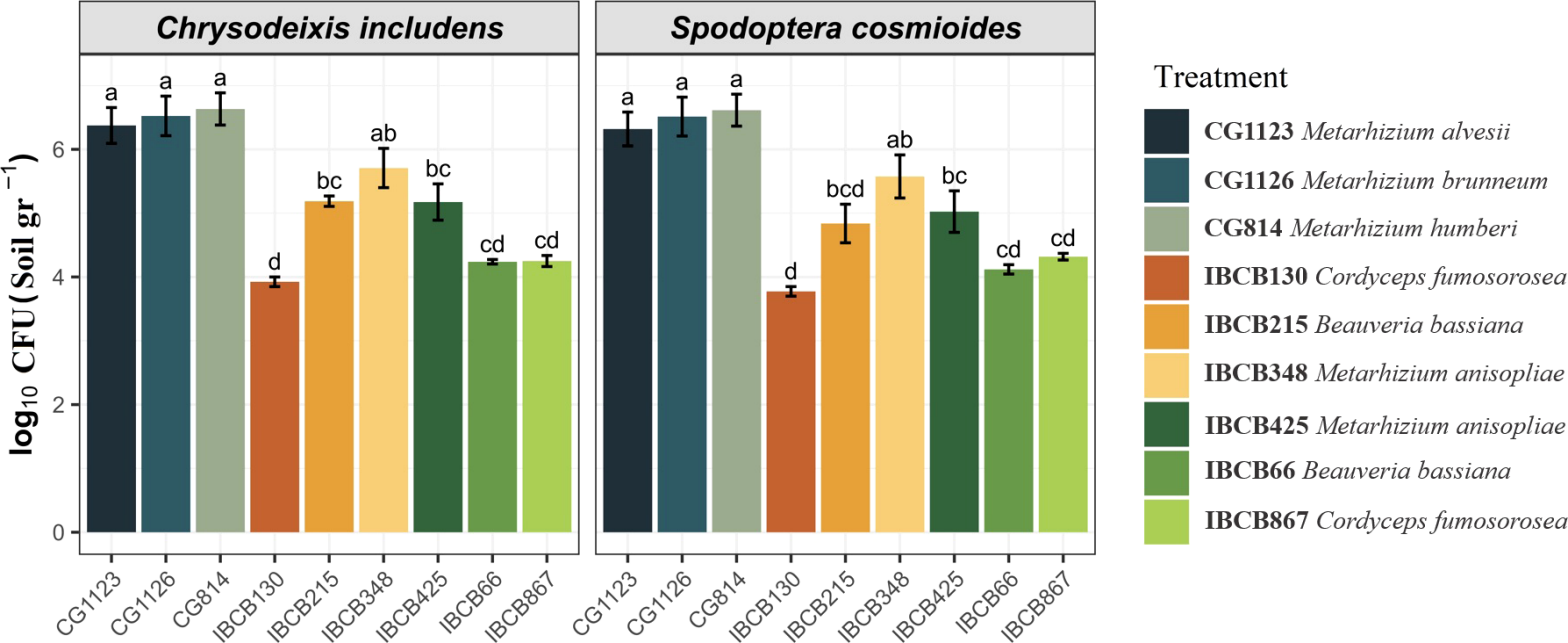
Presence of entomopathogenic fungi in CFU/g soil on peanut plants 30 days after inoculation.

In general, the presence of all entomopathogenic fungi was higher in the soil than in the phyllosphere. For instance, the presence of some *Metarhizium* strains was significantly higher in soil (CG814 7.7 x 10^6^ CFU/g, CG1126 6.2 x 10^6^ CFU/g, CG1123 4.0 x 10^6^ CFU/g and IBCB 348 9.5 x 10^5^ CFU/g), followed by *B. bassiana* IBCB215 (1.6 x 10^5^ CFU/g) and *M. anisopliae IBCB425* (3.1 x 10^5^ CFU/g) (see **Figure 2**).

## Discussion

Most studies of entomopathogenic fungi in agriculture over time have focused on their pathogenicity to insects by spraying formulated products on plants. However, recent studies of the ecological interaction of these fungi in soils and plants are providing a novel alternative for the management of insect pests, such as the potential of the endophytic relationship of these fungi with plants (Bamisile et al., 2018; Vega et al., 2018; Mantzoukas et al., 2022).

The ability to minimize attack from lepidopterous larvae has widely been reported by Vega et al. (2018) for many crops, but to date, no studies have been conducted with peanut plants in this sense.

Our results provide the first report on survivorship and performance parameters for *S. cosmioides* and *C. includens* fed inoculated peanut plants with entomopathogenic fungi (*Metarhizium, Beauveria* and *Cordyceps*).

Fungal endophytes can provide protection for crop plants against insect attack. However, some fungal and plant endophytic associations do not necessarily protect the plant from pest attacks, and only a few species associations function as defensive mutualism (Bamisile et al., 2021).

Several studies investigating the role of endophytes in protecting different crops against lepidopteran species did not measure insect performance parameters; however, they observed a reduction in the damage caused by pests (Jaber, L. R., Enkerli, J. (2017); Bamisile et al., 2018; Vega et al., 2018).

Environmental factors play a vital role in entomopathogenic fungi survival. Generally, entomopathogenic fungi survive well in nature between 10°C and 30°C, and the ideal temperature and higher humidity with frequent water spray observed in our study area could favor the activity of the fungal pathogens, as reported by Mc Coy et al. (1988).

Studying the persistence of entomopathogenic fungal pathogens is essential for their successful application and efficacy in the soil (Enkerli et al., 2004). In our study, the fungus *B. bassiana* was found in stems and leaves, while *Metarhizium* was mostly found within the plant roots and leaves.

Our results are similar to those obtained by Behie et al. (2015), who reported that *B. bassiana* and *Pochonia chlamydosporia* were localized within the stems and leaves, while *Metarhizium* spp. was mostly found within the plant roots.According to Tefera and Vidal (2009), colonization by *B. bassiana* is more widespread in leaves and stems than in roots.

Our study used the soil-drench technique to inoculate the fungi in peanut plants. Russo et al. (2019) demonstrated the effectiveness of three fungal inoculation methods (leaf spray, seed immersion and root immersion) to establish the fungus *B. bassiana* as an endophyte in soybeans, and the leaf spray inoculation method was the most successful.

However, other authors, such as Parsa et al. (2013), obtained good results in colonizing bean plants using the method performed in our study, and according to these authors and Bamisile et al. (2018), the specific outcome of endophytic colonization may depend on the target crop species or variety, the strain or isolate of entomopathogenic fungi species used, and the plant growth conditions.

Our study showed that *Metarhizium* strains had greater endophytic capacity and greater presence in the soil than the other fungi tested. Jaber and Enkerli (2017) described that fungal entomopathogens may colonize particular host plants more efficiently than others, which might consequently influence the level of plant protection by the colonizing fungi.

A limitation of many studies is that they did not measure the consumption of plant material by larvae feeding on plants inoculated with fungi or control plants, which could show possible inhibition or compensatory feeding behavior by larvae. Measuring the leaf area of ​​whole plants is a difficult task, but in our study, we tested a method using peanut plants of the same age and the same number of leaves after trimming, so it was possible to perform a predetermined average of the whole plants.

Our study showed that the foliar consumption of *S. cosmioides* and *C. includens* larvae was drastically lower for some fungal treatments than for the control.In a similar study, Barta (2018) described that *Cameraria ohridella* larvae that fed on horse-chestnut leaves inoculated with *Beauveria* presented leaf areas up to 5 times smaller than the leaf areas of control plants.

Cabezas et al. (2013) demonstrated that *S. cosmioides* larvae consumed a daily average of 5.58 cm² to 15.3 cm² on castor and barbados nut leaves during development from the third instar to the pupal stage, while Bueno et al. (2011) mentioned that *C. includens* larvae can consume average values ​​of 64 cm^2^ to 200 cm^2^ during the larval cycle. These results are similar to our study based on control treatment for both larvae.

We observed that the consumption of larvae that fed on plants with fungus was reduced by approximately 88 cm^2^ for *S. cosmioides* and 43 cm^2^ for *C. includens*.

The weights of *S. cosmioides* and *C. includens* larvae and pupae were significantly different between the fungal treatment groups in our greenhouse trials. Similar results were obtained in some studies.

The authors McGee (2002); Powell et al. (2009); Leckie et al. (2008) found an effect of endophytes on the gained weight of lepidopteran species *H. armigera*, *H. punctigera* and *H. zea*. However, these effects were observed by incorporating fungal broth extracts of the endophytes into artificial diets rather than evaluating effects on insect growth *via* in planta feeding assays.

Some studies similar to ours that utilized in planta feeding assays (Jallow et al., 2004; Jaber and Vidal, 2010) found a significant decrease in the relative growth rate of *H. armigera* larvae feeding on *A. strictum*-inoculated plants, but they did not observe a difference in pupal weights among treatments.

Additionally, Barta (2018) found that *C. ohridella* larvae fed horse chestnut leaves had lower pupal weights than larvae fed control leaves (without fungus). Lopez and Sword (2015) did not observe a difference in pupal and larval weights of *H. zea* larvae feeding on cotton-inoculated plants.

Our results showed significant larval mortality rates for *S. cosmioides* and *C. includens* of the fungal endophyte treatment on peanut plants*;* almost all strains of *Metarhizium* and only one strain *of B. bassiana* in our study showed the highest rates of mortality. However, the strains of *C. fumosorosea* (IBCB 130 and IBCB 867) and *B. bassiana* IBCB66 did not show high rates of larval mortality compared to other fungi.

Other studies showed similar results with different fungal isolates, plants and insects, including the study conducted by Resquín-Romero et al. (2016), who tested the effects of inoculating the fungi *B. bassiana* and *M. brunneum* on alfalfa, tomato and melon plants on *Spodoptera littoralis* larvae.

The results showed that *S. littoralis* fed on inoculated plants had mortalities ranging from 41.7 to 76.6%. Another study by Peña-peña et al. (2015) showed mortality rates of 50% in *Anomala cincta* larvae that fed on corn plants inoculated with *M. pingshaense*.

Mantzoukas et al. (2015) also showed larval mortality rates of 70 to 100% in *Sesamia nonagrioides* feeding on sorghum plants inoculated with the fungi *B. bassiana*, *M. robertsii* and *I. fumosorosea*. Leckie (2002); Powell et al. (2009) and Lopez and Sword (2015) reported a reduction in the damage caused and mortality of *H. zea* in tomato plants after treatment with *B. bassiana*.

Another study by Sánchez-Rodríguez et al. (2018) showed that 30-57% of *S. littoralis* larvae died after consuming endophytically colonized wheat leaves. Klieber and Reineke (2016) additionally recorded 50% mortality of all larval instars and reduced longevity of *Tuta absoluta* larvae fed with *B. bassiana* colonized with tomato leaves.

In this context, according to Bamisile et al. (2018), entomopathogenic fungal endophytes activate the production of plant defense proteins in their colonizing hosts. Thus, the induced systemic responses produced by fungal endophytes are related to the amplification of expressed genes in pathogenesis (Fadiji and Babalola, 2020).

Their capacity to increase the production of important metabolites, proteins and other defense enzymes in peanuts has been demonstrated by Senthilraja et al. (2013) in peanut seedlings inoculated with *B. bassiana* and *Pseudomonas fluorescens*. In another study, a similar combination of *B. bassiana* and *P. fluorescens* strains significantly reduced damage caused by leafminer (*Aproaerema modicella* Deventer) on peanuts (Senthilraja et al., 2010)

Our study demonstrated developmental delay up to the pupal stage for both larvae that fed on peanut plants inoculated with fungi, and the differences in larval delay in fungi treatments relative to the control reached an average of 3 to 5 days. A similar delay in pupation was observed by Jaber and Vidal (2010), who showed that *H. armigera* feeding on endophyte-treated plants in planta trials exhibited slower larval and prepupal developmental times than individuals feeding on control plants.

In contrast, Leckie et al. (2008), using *H.* zea individuals feeding on *B. bassiana* broth extracts incorporated into an artificial diet, showed that larvae feeding on endophyte treatments pupated faster than the control (13 vs. 15 days). Lopez and Sword (2015) also observed any difference in days to pupation of *H. zea* consuming cotton plants inoculated with *B. bassiana* endophyte in greenhouse trials, but they observed that control insects reached the adult stage faster than the *B. bassiana* endophyte treatment. In our study, we did not evaluate the time from pupa to adult stage of insects.

In conclusion, the manipulation of endophytic fungi has the potential to protect plants from insect herbivores. Our study demonstrated for the first time the positive effects of the endophytic entomopathogens *Metarhizium* and *B. bassiana* in peanut plants. We observed negative effects on the survival and development of two herbivorous insect pests, *S. cosmioides* and *C. includens*. Importantly, although these effects were shown in greenhouse trials, more tests need to be done to achieve field conditions using a simple seed treatment inoculation.

Similarly, a variety of other studies have reported the successful manipulation of fungal endophytes in plants with positive effects against lepidopteran insects under greenhouse and field conditions (Vakili, 1990; Bing and Lewis, 1991; Bing and Lewis, 1992a; Bing and Lewis, 1992b; Cherry et al., 1999; Cherry et al., 2004; Powell et al., 2007; Powell et al., 2009; Reddy et al., 2009; Batta, 2013; Castillo Lopez et al., 2014; Lopez and Sword, 2015; Mantzoukas et al., 2015; Qayyum et al., 2015; Shrivastava et al., 2015; Vidal and Jaber, 2015; Klieber and Reineke, 2016; Ramírez-Rodríguez and Sánchez-Peña, 2016; Resquín-Romero et al., 2016; Sánchez-Rodríguez et al., 2018; Silva et al., 2020).

Finally, these results continue to highlight the viability of incorporating the use of fungal endophytes as functional components of integrated pest management (IPM) practices to protect plants from pests. More evaluation under field conditions is necessary to maximize efficacy when trying to incorporate fungal entomopathogens as endophytes within IPM programs.

## Data availability statement

The datasets presented in this study can be found in the online repository: https://repositorio.unesp.br/handle/11449/238519.

## Author contributions

FV and EL contributed to conception and design of the study. FV organized the database. JN and LD performed the statistical analysis. FV wrote the first draft of the manuscript. FV, CR, LR, and NS wrote sections of the manuscript. All authors contributed to the article and approved the submitted version.
